# Neonatal Outcomes Following Selective Serotonin Reuptake Inhibitor Use During Pregnancy

**DOI:** 10.1001/jamanetworkopen.2026.22790

**Published:** 2026-07-13

**Authors:** Layla Aref, Jacob J. Hughey, Sherwin Shirazi, Jennifer M. S. Sucre, Lisa Bastarache

**Affiliations:** 1 Department of Biomedical Informatics, Vanderbilt University Medical Center, Nashville, Tennessee; 2 Department of Pediatrics, Vanderbilt University Medical Center, Nashville, Tennessee; 3 Department of Cell and Developmental Biology, Vanderbilt University, Nashville, Tennessee; 4 Biodevelopmental Origins of Lung Disease (BOLD) Center, Vanderbilt University School of Medicine, Nashville, Tennessee

## Abstract

**Importance:**

Evaluating the safety of selective serotonin reuptake inhibitors (SSRIs) during pregnancy is challenging due to ethical barriers to randomized trials and potential confounding in observational studies.

**Objective:**

To compare neonatal outcomes for pregnancies with SSRI continuation vs those with SSRI discontinuation using a target trial framework.

**Design, Setting, and Participants:**

This cohort study comprised target trials using electronic health records of linked mothers and neonates from a single academic center. The periconception trial examined congenital anomalies with first-trimester exposure, and the early-pregnancy trial evaluated nonanomaly outcomes with first- or second-trimester exposure. Participants were women with singleton pregnancies (2006 to 2022) with SSRI use within the 2 years before conception and at least 1 prenatal visit during the first trimester (periconception trial) or during the first or second trimester (early-pregnancy trial). Data were analyzed from May 2022 to December 2023.

**Exposure:**

SSRI continuation vs discontinuation during pregnancy.

**Main Outcomes and Measures:**

Apgar scores, meconium in amniotic fluid, neonatal intensive care unit (NICU) admission, preterm birth, birth weight, respiratory distress, feeding problems, hypoglycemia, hypothermia, pulmonary hypertension, cesarean delivery, and congenital anomalies.

**Results:**

The early-pregnancy trial included 1014 pregnancies (mean [SD] maternal age, 30.3 [5.4] years). The periconception trial included 807 pregnancies (mean [SD] maternal age, 30.4 [5.5] years). SSRI continuation was associated with lower Apgar scores at 1 minute (mean difference [MD], –0.39; 95% CI, –0.60 to –0.18) and 5 minutes (MD, –0.28; 95% CI, –0.42 to –0.13), and increased risk of meconium in amniotic fluid (odds ratio [OR], 1.73; 95% CI, 1.22 to 2.45). No significant associations were found for congenital anomalies (OR, 1.09; 95% CI, 0.63 to 1.90), though this result should be interpreted with caution given limited sample size. No differences were found for neonatal intensive care unit admission (OR, 1.23; 95% CI, 0.82 to 1.83) or other outcomes.

**Conclusions and Relevance:**

In this cohort study, SSRI continuation during pregnancy was associated with impaired neonatal adaptation but not with severe complications or NICU admission. These findings highlight the importance of appropriate comparator selection to minimize confounding and illustrate how target trial emulation can improve causal inference in studies of medication safety during pregnancy.

## Key Points

**Question:**

Is continuing SSRI treatment during pregnancy associated with adverse neonatal outcomes compared with discontinuation?

**Findings:**

In this cohort study using a target trial emulation framework and electronic health records from 1014 pregnancies, compared with discontinuation, continuation of SSRI treatment during pregnancy was associated with neonatal adaptation symptoms, including lower Apgar scores. No statistically significant associations were observed with life-threatening complications, neonatal intensive care unit admission, or major congenital anomalies.

**Meaning:**

By restricting to individuals with prepregnancy SSRI use and minimizing bias and confounding, this study suggests that SSRI continuation during pregnancy may be associated with transient, but not severe, neonatal outcomes.

## Teaser

This cohort study uses a target trial emulation approach to evaluate neonatal outcomes for pregnancies with continuation of selective serotonin reuptake inhibitors (SSRIs) vs discontinuation.

## Introduction

Over a lifetime, depression will affect approximately 26% of women.^1^ Selective serotonin reuptake inhibitors (SSRIs) are often prescribed as first-line treatment, and a considerable number of women continue usage during part or all of pregnancy.^2,3,4,5^ As small lipophilic molecules, SSRIs can cross the placenta,^6,7^ raising concerns about potential adverse effects on neonatal health. However, despite extensive research, isolating the risk of prenatal exposure to SSRIs remains challenging.^8^

Randomized clinical trials (RCTs) are the gold standard for establishing drug safety. However, conducting RCTs during pregnancy poses significant and often insurmountable ethical challenges.^9^ As a result, much of what is known about the risks of SSRIs during pregnancy comes from retrospective observational studies,^10,11^ which have produced varying and conflicting findings.^12,13,14^ This variability may stem from study designs that introduce bias through confounding, inappropriate comparator groups, or exposure misclassification.

Through modern statistical methods, we can emulate traditional drug safety trials with observational data. Target trial emulation is a framework that uses observational data to emulate a hypothetical RCT by explicitly defining the key components of an ideal trial and replicating them as closely as possible within the observational dataset, making any deviations explicit.^15,16^ This framework provides an organizing principle for causal inference methods and helps mitigate common biases observed in retrospective cohort studies.

Here, we applied a target trial framework to a database that links electronic health records (EHRs) of mothers and newborns to evaluate neonatal outcomes following SSRI exposure during pregnancy. Using this approach, we assessed a range of neonatal outcomes, including measures of neonatal adaptation and more severe complications. We demonstrate how the target trial framework addresses biases found in more conventional analytic approaches and show how EHR linkage enhances data completeness and the range of variables available for study. Taken together, these findings provide a more nuanced understanding of SSRI use during pregnancy and underscore the importance of rigorous study design in evaluating medication safety.

## Methods

### Data Source

Our cohort included pregnant women and their offspring via the Mom-Baby Dyad Database (MBdb). This resource links records for 77 813 mother-baby dyads (58 912 mothers and 76 074 unique pregnancies). All MBdb patients were seen at Vanderbilt University Medical Center (VUMC), an academic tertiary referral center in the southeastern US, from 2006 to 2022. The MBdb includes EHR-extracted data elements, including estimated date of conception (EDC), estimated gestational age (EGA), diagnoses, test results, and medications in the prepregnancy and perinatal period. Each pregnancy is defined as starting at EDC and ending with offspring date of birth (DOB). Because linkage requires a live-born infant, analyses are restricted to live births and reflect associations conditional on survival to delivery. Data elements were annotated with temporal markers to indicate EGA for those occurring intrapartum and days to EDC for those occurring prepregnancy. eTable 1 in Supplement 1 defines EHR variables. This study was reviewed by the VUMC institutional review board with a non–human participants determination and therefore was not required to collect informed consent. We used MBdb to emulate a target trial protocol, as described in the subsequent section. Reporting followed Strengthening the Reporting of Observational Studies in Epidemiology (STROBE) reporting guidelines for comparative effectiveness research.^17^

### Eligibility Criteria

#### Target Trial Protocol

Pregnant women with singleton pregnancies in the years 2006 to 2022 who were on a treatment course of SSRIs at any time during the 2 years before EDC and received prenatal care at VUMC were included. To account for the effects of exposures at different pregnancy and/or fetal development stages, we defined 2 target trial versions. In the early-pregnancy trial, pregnant women remained eligible until end of the second trimester to assess noncongenital neonatal outcomes. In the periconception trial, pregnant women remained eligible until end of the first trimester (when organogenesis occurs) to assess congenital anomalies.

#### Protocol Emulation

We identified SSRI-exposed women within the 2 years before pregnancy (see eAppendix 1 in Supplement 2). For data completeness, we required at least 1 prenatal visit within the relevant eligibility window. We excluded pregnancies with missing body mass index (BMI) or molecularly confirmed genetic diagnoses (eg, trisomy 21) in the infant (periconception trial).

### Treatment Strategies

#### Target Trial Protocol

In the continuation group, participants continued exposure to SSRI initiated before pregnancy until the end of the enrollment window (end of the first trimester for the periconception trial and end of the second trimester for the early-pregnancy trial). In the discontinuation group, participants discontinued SSRIs before EDC and had no SSRI exposure during pregnancy.

To maintain adherence to the specified treatment strategies, participants initiating non-SSRI antidepressants or anxiolytics during pregnancy were excluded (eAppendix 2 in Supplement 2). This reflects a per-protocol design, estimating the effect of continuing SSRIs without alternative treatments.

#### Protocol Emulation

We defined the continuation group having either (1) an inpatient or prescription record of SSRIs between EDC and the end of the enrollment period, or (2) a medication list entry during that same window combined with inpatient or prescription SSRI use during hospital stay for delivery (eAppendix 1 in Supplement 2). This inclusion assumes that SSRI administration at delivery reflects continuation of treatment initiated earlier in pregnancy rather than reinitiation. We excluded pregnancies with any record of non-SSRI antidepressants or anxiolytics during pregnancy (eAppendix 2 in Supplement 2).

The discontinuation group included participants with no recorded SSRI exposure during pregnancy. To minimize misclassification, we excluded pregnancies with inpatient or prescription SSRI exposure during delivery, SSRI prescription within 90 days before EDC, or any non-SSRI antidepressant or anxiolytic records during pregnancy (eAppendix 2 in Supplement 2). To validate our treatment assignment algorithm, we manually reviewed 30 randomly selected EHRs from each group to compute the positive predictive value (PPV).

### Assignment Procedures

In the target trial protocol, eligible women are randomly assigned to either strategy at baseline. For the protocol emulation, we assigned each eligible woman to the treatment strategy compatible with their data, emulating randomization using the following baseline variables: maternal age at EDC, delivery year, EHR race, insurance type, marital status, gravida, smoking status, BMI at EDC, Charlson comorbidity score,^18,19^ number of health care visits, substance use disorder, and number of dates with depression diagnosis as a proxy for depression severity (eTable 1 in Supplement 1 for defined variables). EHR race and ethnicity, as determined from patient or caregiver report in the EHR, was included due to its association with differences in health outcomes and access to care.^20^ Patient Health Questionnaire-9 (PHQ-9) scores were considered a measure of depression severity^21^; however, due to low availability (10% of records), they were not used in the analysis.

Ideally, baseline variables are measured at treatment assignment to avoid posttreatment bias. However, precise treatment start dates are difficult to determine in EHRs. We therefore used EDC as a proxy for baseline. Covariates based on diagnostic or utilization data were defined using a 2-year lookback window from EDC (see eTable 1 in Supplement 1).

### Outcomes

#### Target Trial Protocol

We selected outcomes informed by previous studies^22,23,24^ as well as those indicating life-threatening conditions and symptoms of poor neonatal transition to extrauterine life. Outcomes for the early-pregnancy trial include gestational age, birth weight, preterm birth (gestational age <37 weeks), neonatal intensive care unit (NICU) admission, Apgar scores at 1 and 5 minutes, cesarean delivery, respiratory distress syndrome, hypoglycemia, hypothermia, feeding problems, persistent pulmonary hypertension of the newborn (PPHN), and meconium staining. The outcomes for the periconception trial include major congenital malformations, multiple congenital malformations, and congenital heart defects.

#### Protocol Emulation

Outcomes were identified using *International Classification of Diseases, Ninth Revision (ICD-9)* and *Tenth Revision (ICD-10) *diagnostic codes or extracted from the first neonatal discharge summary (eTable 1 in Supplement 1). Outcomes related to congenital anomalies were initially identified using PhecodeX^25^ grouping for standardized mappings of *ICD* codes.

The literature reports positive predictive values as low as 18% for congenital codes.^26^ To address this, we manually reviewed EHRs of all infants with 1 or more qualifying congenital anomaly codes for validation (see eAppendix 3 in Supplement 2 and eTable 2 in Supplement 1 for outcome definitions and procedures).

### Follow-Up Period

In the target trial, follow-up starts at treatment assignment and ends at either the occurrence of the outcome of interest, 30 days after birth (for congenital anomalies), or 7 days after birth (for other outcomes), whichever occurred earliest. The follow-up for the protocol emulation was the same as the target trial.

### Causal Contrast of Interest

The target trial estimated the per-protocol effect. The protocol emulation estimated an observational analog of the per-protocol effect (ie, the protocol of continuing SSRI treatment during pregnancy vs discontinuing).

### Statistical Analysis

In the target trial protocol, we used linear regression to estimate mean differences for continuous outcomes and logistic regression to estimate odds ratios (ORs) for binary outcomes, comparing SSRI continuation vs discontinuation. Models were adjusted for offspring sex with additional adjustment for gestational age when modeling birth weight. Effect estimates and 95% CIs were emphasized over statistical significance. When reported, statistical significance was assessed using 2-sided hypothesis tests with α = .05, and results with *P* < .05 were considered statistically significant.

For the protocol emulation, the analysis plan was the same as the target trial, except that we used inverse probability weighting (IPW) with overlap weights to adjust for baseline covariates (listed under assignment procedures).^27^ We estimated 95% CIs using robust variance. These results were compared with the unadjusted analysis.

We also compared our results with scenarios where the control group is not carefully selected and validated. To achieve this, we defined a second control group, referred to as the never-exposed group, consisting of singleton pregnancies with no documented antidepressant exposure in the 2 years before and during pregnancy. This analysis was restricted to early-pregnancy outcomes, comparing the never-exposed group with the continuation group from the early-pregnancy cohort. To ensure comparability, we applied selected eligibility criteria: at least 1 prenatal visit in the first or second trimester, nonmissing BMI, no use of non-SSRI antidepressants or anxiolytics during pregnancy, at delivery, or in the 2 years prior, and at least 1 health care encounter in the 2 years before pregnancy to confirm opportunity for documenting exposure. Regression analyses were conducted to compare outcomes between the never-exposed and continuation groups.

Three sensitivity analyses were conducted. First, we assessed the outcomes of narrowing the prepregnancy exposure window from 2 years to 1 year, repeating the emulation study using a 1-year lookback period. Second, to address potential selection bias from excluding women who switched to or were coprescribed a non-SSRI antidepressant or anxiolytic during pregnancy, we applied stabilized inverse probability of censoring weights (IPCW) estimated from the same baseline covariates as the primary propensity score model, and multiplied these by the primary IPW weights.^28^ Third, we evaluated the outcomes of within-mother correlation due to repeated pregnancies using cluster-robust standard errors.

All statistical analyses were performed using R (version 4.2.2). Data were analyzed from May 2022 to December 2023.

## Results

Among the 1014 pregnancies included in the early-pregnancy trial, the mean (SD) maternal age was 30.3 (5.4) years. A total of 110 pregnancies (10.8%) occurred among Black individuals, 29 (2.9%) among White Hispanic individuals, and 796 (78.5%) among White non-Hispanic individuals. Among the 807 pregnancies included in the periconception trial, the mean (SD) maternal age was 30.4 (5.5) years, with 80 pregnancies (9.9%) among Black individuals, 22 (2.7%) among White Hispanic individuals, and 641 (79.4%) among White non-Hispanic individuals. In the early-pregnancy comparison of SSRI continuation vs never exposure (21 923 pregnancies), the mean (SD) maternal age was 29.7 (5.6) years. A total of 4456 pregnancies (20.3%) occurred among Black individuals, 1110 (5.1%) among White Hispanic individuals, and 13 315 (60.7%) among White non-Hispanic individuals.

**Figure 1.**
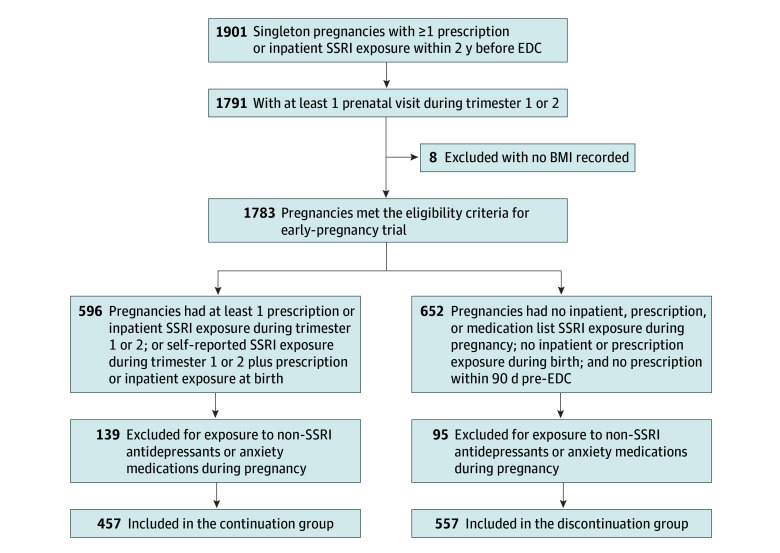
Flowchart Showing Early-Pregnancy Target Trial Emulation Cohort Assembly BMI indicates body mass index; EDC, estimated date of conception; SSRI, selective serotonin reuptake inhibitor.

**Figure 2.**
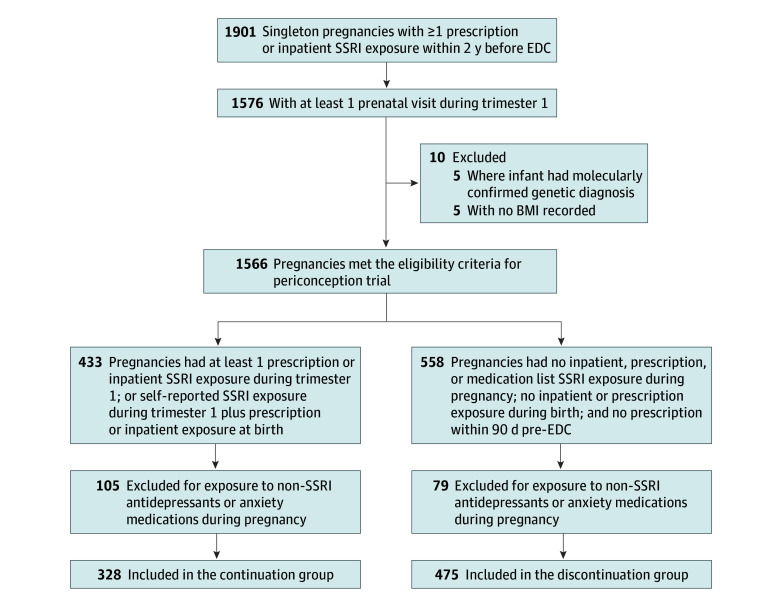
Flowchart Showing Periconception Target Trial Emulation Cohort Assembly BMI indicates body mass index; EDC, estimated date of conception; SSRI, selective serotonin reuptake inhibitor.

Among the 76 074 pregnancies in the MBdb, 38 797 (51.0%) lacked any encounter with VUMC in the 2 years before pregnancy. We identified 1901 singleton pregnancies with documentation of SSRI exposure during the 2 years before EDC. A total of 1783 met the eligibility criteria for the early-pregnancy trial, and 1566 for the periconception trial. Within the early-pregnancy trial cohort, 457 pregnancies were assigned to the continuation group and 557 pregnancies to the discontinuation group (Figure 1). Within the periconception trial, 328 pregnancies were assigned to the continuation group, and 479 pregnancies to the discontinuation group (Figure 2). Manual EHR review of treatment strategies in the early pregnancy trial revealed a PPV of 94% for the continuation group and a PPV of 85% for the discontinuation group.

Several significant differences were observed between continuation and discontinuation groups, including maternal age, race, insurance status, and marital status, with 10 of 14 variables significantly different in the early pregnancy cohort and 7 in the periconception cohort. Importantly, the number of visits during the 2 years before pregnancy was similar between groups, suggesting comparable levels of engagement with the VUMC health system. There were more substantial differences between the continuation and never-exposed groups, particularly in variables indicative of health and health care utilization (Table).

**Table.** Comparison of Baseline Variables Across Groups Within Different Cohorts

Characteristics	Patients, No. (%)			Test statistic	*P* value
	Continuation	Control group	Full cohort		
Early pregnancy cohort					
Continuation vs discontinuation	n = 457	n = 557	n = 1014	NA	NA
Age, mean (SD), y	31.1 (5.1)	29.6 (5.6)	30.3 (5.4)	4.6^a^	<.001
Year of delivery					
2006-2009	21 (4.6)	33 (5.9)	54 (5.3)	9.91^b^	.04
2010-2012	55 (12)	60 (10.8)	115 (11.3)		
2013-2015	45 (9.8)	88 (15.8)	133 (13.1)		
2016-2018	84 (18.4)	104 (18.7)	188 (18.5)		
2019-2022	252 (55.1)	272 (48.8)	524 (51.7)		
EHR race					
Black	32 (7.0)	78 (14.0)	110 (10.8)	33.46^b^	<.001
White Hispanic	15 (3.3)	14 (2.5)	29 (2.9)		
White non-Hispanic	392 (85.8)	404 (72.5)	796 (78.5)		
Other^c^	17 (3.7)	59 (10.6)	76 (7.5)		
Unknown	1 (0.2)	2 (0.4)	3 (0.3)		
Insurance					
Commercial	337 (73.7)	340 (61.0)	677 (66.8)	19.49^b^	<.001
Medicare or Medicaid	110 (24.1)	190 (34.1)	300 (29.6)		
Other or unknown	10 (2.2)	27 (4.8)	37 (3.6)		
Marital status					
Married	329 (72.0)	328 (58.9)	657 (64.8)	20.31^b^	<.001
Single	86 (18.8)	168 (30.2)	254 (25.0)		
Other or unknown	42 (9.2)	61 (11.0)	103 (10.2)		
Gravida					
1	148 (32.4)	144 (25.9)	292 (28.8)	4.91^b^	.027
>1	309 (67.6)	413 (74.1)	722 (71.2)		
Unknown	0	0			
Smoking					
Ever smoked	128 (28.0)	197 (35.4)	325 (32.1)	7.06^b^	.03
Never smoked	321 (70.2)	347 (62.3)	668 (65.9)		
Unknown	8 (1.8)	13 (2.3)	21 (2.1)		
BMI, mean (SD)	28.9 (7.6)	28.1 (7.0)	28.4 (7.3)	1.82^a^	.07
Charlson Comorbidity score	0.3 (0.9)	0.2 (0.8)	0.3 (0.9)	1.57^a^	.12
Visits (during 2 y before EDC), mean (SD)	19.1 (15.9)	18.5 (17.5)	18.8 (16.8)	0.57^a^	.57
Dates with depression diagnosis (during 2 y before EDC), mean (SD)	0.6 (1.5)	0.4 (1.3)	0.5 (1.4)	2.09^a^	.04
Dates with anxiety diagnosis (during 2 y before EDC), mean (SD)	0.7 (1.6)	0.4 (0.9)	0.5 (1.3)	4.44^a^	<.001
Substance use disorder (during 2 y before EDC)					
Yes	16 (3.5)	38 (6.8)	54 (5.3)	4.85^b^	.03
No	441 (96.5)	519 (93.2)	960 (94.7)		
Substance use disorder during pregnancy					
Yes	27 (5.9)	48 (8.6)	75 (7.4)	2.31^b^	.13
No	430 (94.1)	509 (91.4)	939 (92.6)		
Periconception cohort					
Continuation vs discontinuation	n = 328	n = 479	n = 807	NA	NA
Age, mean (SD), y	31.4 (5.2)	29.8 (5.6)	30.4 (5.5)	4.07^a^	<.001
Year of delivery					
2006-2009	16 (4.9)	28 (5.8)	44 (5.5)	5.16^b^	.27
2010-2012	47 (14.3)	51 (10.6)	98 (12.1)		
2013-2015	39 (11.9)	76 (15.9)	115 (14.3)		
2016-2018	68 (20.7)	89 (18.6)	157 (19.5)		
2019-2022	158 (48.2)	235 (49.1)	393 (48.7)		
EHR race					
Black	20 (6.1)	60 (12.5)	80 (9.9)	18.91^b^	<.001
White Hispanic	12 (3.7)	10 (2.1)	22 (2.7)		
White non-Hispanic	280 (85.4)	361 (75.4)	641 (79.4)		
Other^c^	15 (4.6)	46 (9.6)	61 (7.6)		
Unknown	1 (0.3)	2 (0.4)	3 (0.4)		
Insurance					
Commercial	252 (76.8)	315 (65.8)	567 (70.3)	12.79^b^	.002
Medicare or Medicaid	70 (21.3)	142 (29.6)	212 (26.3)		
Other or unknown	6 (1.8)	22 (4.6)	28 (3.5)		
Marital status					
Married	242 (73.8)	293 (61.2)	535 (66.3)	15.48^b^	<.001
Single	57 (17.4)	138 (28.8)	195 (24.2)		
Other or unknown	29 (8.8)	48 (10.0)	77 (9.5)		
Gravida					
1	117 (35.7)	134 (28.0)	251 (31.1)	5.03^b^	.03
>1	211 (64.3)	345 (72.0)	556 (68.9)		
Unknown	0	0			
Smoking					
Ever smoked	79 (24.1)	161 (33.6)	240 (29.7)	9.86^b^	.007
Never smoked	245 (74.7)	308 (64.3)	553 (68.5)		
Unknown	4 (1.2)	10 (2.1)	14 (1.7)		
BMI, mean (SD)	28.7 (7.9)	28.0 (7.0)	28.3 (7.3)	1.28^a^	.20
Charlson Comorbidity score	0.3 (0.8)	0.2 (0.7)	0.2 (0.8)	1.5^a^	.14
Visits (during 2 y before EDC), mean (SD)	18.2 (15.5)	18.7 (18.3)	18.5 (17.2)	−0.43^a^	.67
Dates with depression diagnosis (during 2 y before EDC), mean (SD)	0.6 (1.7)	0.4 (1.4)	0.5 (1.5)	1.98^a^	.05
Dates with anxiety diagnosis (during 2 y before EDC), mean (SD)	0.7 (1.5)	0.3 (0.9)	0.5 (1.2)	3.69^a^	<.001
Substance use disorder (during 2 y before EDC)					
Yes	8 (2.4)	24 (5.0)	32 (4.0)	2.74^b^	.10
No	320 (97.6)	455 (95.0)	775 (96.0)		
Substance use disorder during pregnancy					
Yes	13 (4.0)	32 (6.7)	45 (5.6)	2.24^b^	.14
No	315 (96.0)	447 (93.3)	762 (94.4)		
Early pregnancy cohort (secondary comparison)^d^					
Continuation vs never exposed	n = 457	n = 21 466	n = 21 923	NA	NA
Age, mean (SD), y	31.1 (5.1)	29.7 (5.6)	29.7 (5.6)	5.97^a^	<.001
Year of delivery					
2006-2009	21 (4.6)	2946 (13.7)	2967 (13.5)	104.75^b^	<.001
2010-2012	55 (12.0)	3355 (15.6)	3410 (15.6)		
2013-2015	45 (9.8)	3692 (17.2)	3737 (17.0)		
2016-2018	84 (18.4)	4201 (19.6)	4285 (19.5)		
2019-2022	252 (55.1)	7272 (33.9)	7524 (34.3)		
EHR race					
Black	32 (7.0)	4424 (20.6)	4456 (20.3)	125.14^b^	<.001
White Hispanic	15 (3.3)	1095 (5.1)	1110 (5.1)		
White non-Hispanic	392 (85.8)	12 923 (60.2)	13 315 (60.7)		
Other^c^	17 (3.7)	2769 (12.9)	2786 (12.7)		
Unknown	1 (0.2)	255 (1.2)	256 (1.2)		
Insurance					
Commercial	337 (73.7)	12 633 (58.9)	12 970 (59.2)	42.14^b^	<.001
Medicare or Medicaid	110 (24.1)	7755 (36.1)	7865 (35.9)		
Other or unknown	10 (2.2)	1078 (5.0)	1088 (5.0)		
Marital status					
Married	329 (72.0)	13 711 (63.9)	14 040 (64.0)	12.98^b^	.002
Single	86 (18.8)	5360 (25.0)	5446 (24.8)		
Other or unknown	42 (9.2)	2395 (11.2)	2437 (11.1)		
Gravida					
1	148 (32.4)	5689 (26.5)	5837 (26.6)	9.53^b^	.009
>1	309 (67.6)	15 689 (73.1)	15 998 (73.0)		
Unknown	0	88 (0.4)	88 (0.4)		
Smoking					
Ever smoked	128 (28.0)	4411 (20.5)	4539 (20.7)	21.89^b^	<.001
Never smoked	321 (70.2)	16 030 (74.7)	16 351 (74.6)		
Unknown	8 (1.8)	1025 (4.8)	1033 (4.7)		
BMI, mean (SD)	28.9 (7.6)	27.3 (6.6)	27.3 (6.7)	4.54^a^	<.001
Charlson Comorbidity score	0.3 (0.9)	0.1 (0.5)	0.1 (0.5)	4.56^a^	<.001
Visits (during 2 y before EDC), mean (SD)	19.1 (15.9)	9.7 (9.7)	9.9 (9.9)	12.59^a^	<.001
Dates with depression diagnosis (during 2 y before EDC), mean (SD)	0.6 (1.5)	0.0 (0.1)	0.0 (0.3)	8.26^a^	<.001
Dates with anxiety diagnosis (during 2 y before EDC), mean (SD)	0.7 (1.6)	0.0 (0.2)	0.0 (0.3)	9.75^a^	<.001
Substance use disorder (during 2 y before EDC)					
Yes	16 (3.5)	174 (0.8)	190 (0.9)	34.64^b^	<.001
No	441 (96.5)	21 292 (99.2)	21 733 (99.1)		
Substance use disorder during pregnancy					
Yes	27 (5.9)	523 (2.4)	550 (2.5)	20.65^b^	<.001
No	430 (94.1)	20 943 (97.6)	21 373 (97.5)		

Abbreviations: BMI, body mass index (calculated as weight in kilograms divided by height in meters squared); EDC, estimated date of conception; EHR, electronic health record; NA, not applicable.

^a^ *T* statistic.

^b^ χ^2^ statistic.

^c^ The EHR race category of other includes American Indian or Alaskan Native, Asian, Native Hawaiian or Pacific Islander, and other.

^d^ This section represents a secondary comparison within the early-pregnancy cohort, comparing continuation vs never-exposed individuals using a nonuser reference group, as described in the Statistical Analysis section of the Methods.

Information on birth outcomes was recorded for almost all women in both early-pregnancy and periconception trials (>99.9%). In addition, no pregnancies were lost to follow-up due to the requirement for continuous enrollment through delivery.

In the periconception trial, we identified 148 infants as possibly having congenital anomalies by *ICD-9* and *ICD-10 *codes. Upon EHR review, 66 of these infants were confirmed to have congenital anomalies (PPV, 45%). Of the 807 infants included in the periconception trial, 60 (7.4%) had a congenital anomaly, 15 (1.9%) had multiple anomalies, and 24 (3.0%) had congenital anomalies of the heart (eTable 3 in Supplement 1).

SSRI continuation was associated with lower Apgar scores at 1 minute (mean difference [MD], –0.39; 95% CI, –0.60 to –0.18) and 5 minutes (MD, –0.28; 95% CI, –0.42 to –0.13), and increased risk of meconium fluid (OR, 1.73; 95% CI, 1.22 to 2.45). No significant associations were found for congenital anomalies (OR, 1.09; 95% CI, 0.63 to 1.90), though this result should be interpreted with caution given limited sample size. No differences were found for neonatal intensive care unit admission (OR, 1.23; 95% CI, 0.82 to 1.83) or other outcomes.

**Figure 3.**
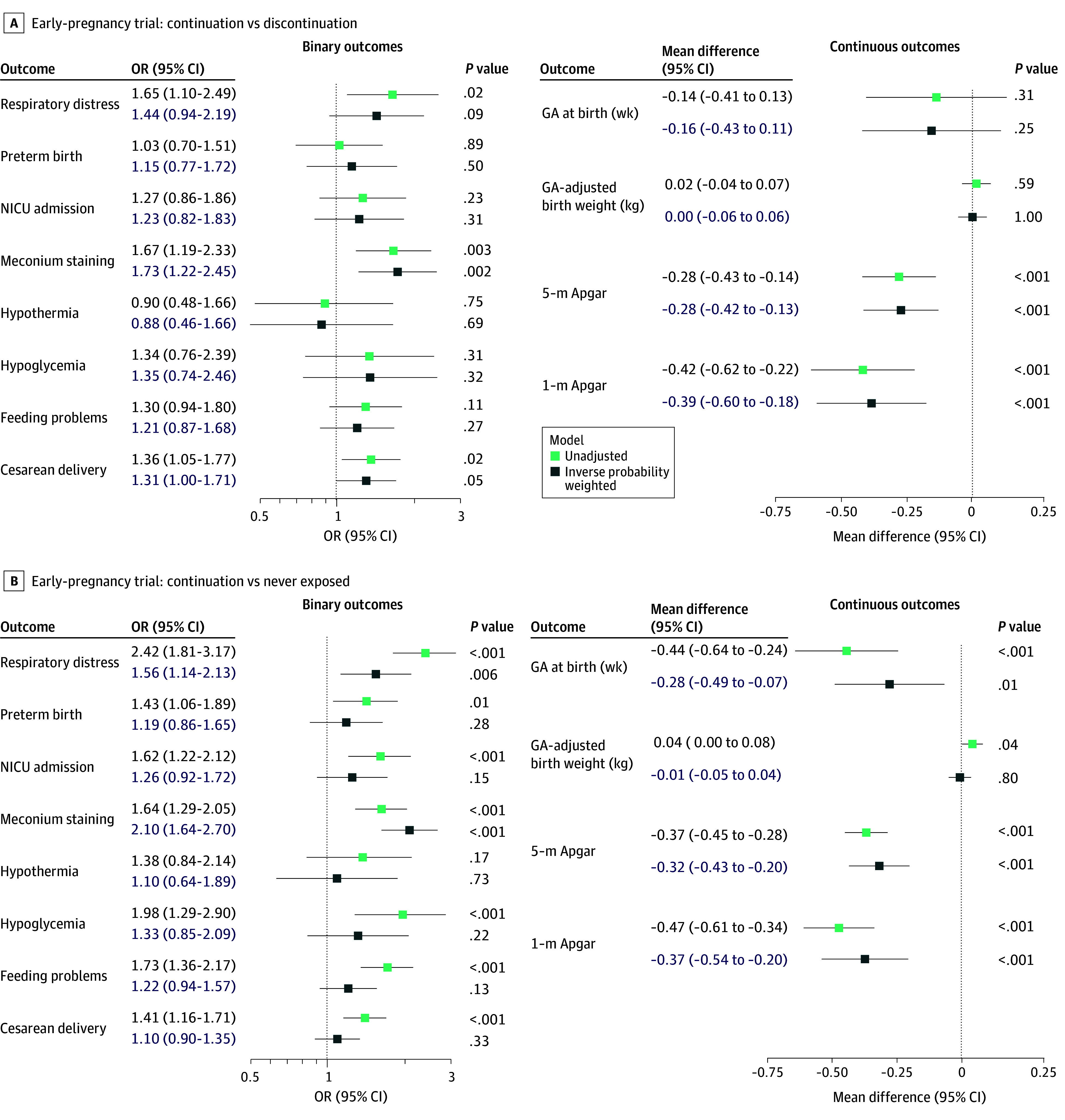
Dot Plot Showing Odds Ratios (ORs) and Mean Differences for Neonatal Outcomes in the Early-Pregnancy Trial A, Continuation vs discontinuation (reference). B, Continuation vs never-exposed (reference). Panels are shown together to facilitate comparison across analytic approaches. Persistent pulmonary hypertension of the newborn was excluded due to wide confidence intervals, which affected interpretability of the plot. eFigures 1 and 2 in Supplement 2 present Apgar score results using categorical measures. GA indicates gestational age; NICU, neonatal intensive care unit.

In the unadjusted analysis for the early-pregnancy trial (comparing continuation and discontinuation), continuing SSRIs during pregnancy was significantly associated with increased risk of meconium staining, respiratory distress, cesarean delivery, and lower 1- and 5-minute Apgar scores. These associations remained largely unchanged after adjusting for baseline covariates using IPW, except for respiratory distress and cesarean delivery, which were not nominally significant after adjustment. (Figure 3A; eTable 4 in Supplement 1).

**Figure 4.**
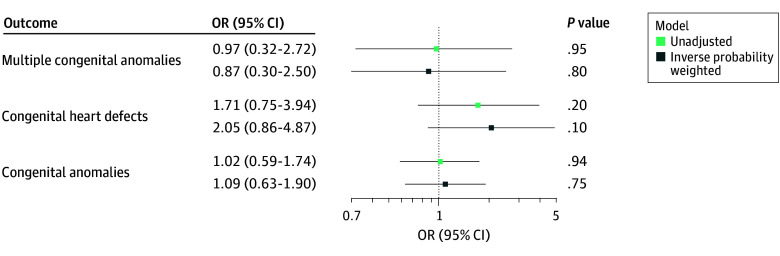
Dot Plot Showing Odds Ratios (OR) of Outcomes in the Periconception Trial Figure shows continuation vs discontinuation (reference).

In unadjusted analysis comparing the continuation group in the early-pregnancy trial to the never-exposed group, exposure to SSRIs was associated with 11 of the 13 outcome variables. These associations were markedly reduced or fully disappeared after applying IPW (Figure 3B; eTable 6 in Supplement 1). In the periconception trial, neither the unadjusted nor IPW analyses showed an association with the 3 congenital anomaly outcomes assessed (Figure 4; eTable 5 in Supplement 1).

In the first sensitivity analysis, reducing the exposure window to 1 year nearly halved the cohort size. Although this adjustment did not change the direction or significantly alter the magnitude of effect estimates, the association for meconium staining was no longer nominally significant (eTables 7 and 8 and eFigures 3 and 4 in Supplement 2).

In the second sensitivity analysis, applying IPCW to account for exclusion of women who switched to or were coprescribed a non-SSRI antidepressant or anxiolytic produced results largely consistent with the primary analysis. The association with meconium staining was attenuated and was not significant, while respiratory distress, which was not significant in the primary analysis, reached statistical significance (eTables 9 and 10 and eFigures 5 and 6 in Supplement 2).

A total of 60 of 1014 pregnancies (5.9%) in the early-pregnancy cohort and 41 of 807 (5.1%) in the periconception cohort were from mothers with repeat pregnancies. Rerunning weighted models with cluster-robust standard errors to account for within-mother correlation did not materially affect standard errors or conclusions.

## Discussion

In this cohort study with a target trial emulation design using EHR data, we found that continuation of SSRIs during pregnancy was associated with an increased risk of meconium-stained amniotic fluid and lower 1- and 5-minute Apgar scores. However, no statistically significant associations were observed for the remaining 13 outcomes examined. These findings provide new insights into our understanding of the safety of SSRIs during pregnancy, a question unlikely to be resolved through randomized trials due to ethical and logistical constraints. By explicitly defining the target trial protocol and its emulation, we aimed to enhance transparency and reproducibility, enabling others to assess and replicate our approach. Importantly, aligning with a trial framework helped mitigate common biases in observational studies, such as ensuring that confounders were measured at or before baseline to avoid posttreatment bias.

Our findings strengthen prior evidence linking in utero SSRI exposure to lower Apgar scores.^29^ This association aligns with clinical reports of delayed neonatal adaptation to extrauterine life, marked by symptoms such as poor muscle tone, jitteriness, weak cry, and respiratory distress, which are usually transient.^30,31^ Reproducing these findings within a target trial framework addresses limitations of earlier small observational studies, which often lacked robust adjustment for confounding factors related to maternal mental health.^32,33^ The association with meconium staining was attenuated and not significant after accounting for potential selection bias from medication switching and should be interpreted with caution. We did not find evidence of an association between maternal SSRI continuation and congenital malformations. While this aligns with prior research, our limited sample size and the rarity of these outcomes preclude definitive conclusions, particularly regarding congenital heart defects.^34,35^ More recent, larger population-based studies indicate that weak associations with specific SSRIs or specific malformations cannot be ruled out.^36,37^

In contrast, our results challenge previous research suggesting an association between maternal SSRI exposure and earlier gestational age at birth.^29,38,39^ While this association was observed when using a control group who was never exposed to SSRIs, it was no longer significant when the control group was restricted to women with a history of SSRI exposure before pregnancy. Although our sample size limits the ability to definitively exclude an association, attenuation of this association under more rigorous modeling suggests that prior findings may have been influenced by unaccounted confounders.

Overall, this study highlights the challenges of confounding in observational studies and the importance of appropriate comparators to minimize bias. In our cohort, individuals who used SSRIs during pregnancy differed markedly from never-exposed individuals across demographic and health-related variables. These differences led to associations that did not replicate in the more rigorously designed target trial emulation using a more comparable discontinuation group, even after adjustment for baseline characteristics, emphasizing the critical role of comparator selection in reducing residual confounding. Additionally, this study shows the potential of EHR data to provide complementary insights to structured data elements like *ICD-9 *and *ICD-10* codes.

As SSRIs are commonly prescribed during pregnancy, there is a need to understand the possible effects on offspring. Historically, when SSRIs were implicated in the risk of life-threatening PPHN, many women chose to discontinue them during pregnancy until larger studies showed that this association was not as strong (2009-2015).^12,14^ Notably, our data reflected this practice change, with fewer women continuing SSRIs during pregnancy from 2013 to 2015. While our study is not intended to dictate clinical practice, our data suggest that SSRIs are not associated with an increase in severe complications. Although we noted an association with slightly lower Apgar scores, a lower Apgar score in the setting of no other adverse complications (eg, NICU admission) does not suggest significant harm in the short or long term. The possible improvement in immediate Apgar scores must be weighed against any potential negative impact on long-term parent and child mental health outcomes before deciding to discontinue SSRI treatment during pregnancy.

### Limitations

SSRI exposure was defined using prescription records, which do not confirm adherence or continuous exposure throughout the third trimester. Prior studies report SSRI discontinuation in late pregnancy, though the estimated rates vary.^2,3^ Findings related to delayed neonatal adaptation should be interpreted with caution, as exposure misclassification may underestimate outcomes associated with late-pregnancy exposure.^31,40^

The requirement for continuous enrollment through delivery excluded miscarriages. Therefore, findings reflect associations among live births and do not account for pregnancy loss as a competing event. Exposure classification relied on medication records accrued during pregnancy, with delivery records used only to corroborate self-reported continuation when prescription documentation was incomplete, which may introduce timing-related bias. Restricting analyses to individuals with prepregnancy SSRI use may limit generalizability.

While the target trial design and IPW address many sources of confounding, residual confounding remains possible. In particular, PHQ-9 scores were available for only about 10% of patients, limiting adjustment for depression severity, a known limitation in routine clinical data.^41^ Although we adjusted for proxy measures, residual confounding by indication may persist.

Multiple neonatal outcomes were evaluated without formal adjustment for multiple comparisons, increasing the risk of type I error. However, outcomes were prespecified based on prior literature, and results should be interpreted in the context of the overall pattern of findings and existing evidence.

Additionally, stringent inclusion criteria reduced our sample size, limiting power to detect associations with rare outcomes, including congenital anomalies, and preventing stratified analyses by SSRI type and/or dosage. Larger population-based studies are needed to evaluate associations between SSRI exposure and rare outcomes.

## Conclusions

In this cohort study of SSRI continuation vs discontinuation during pregnancy, continuation was associated with delayed neonatal adaptation after birth, but not with major anomalies or severe complications. Considering the potential for delayed adaptation after birth, our study supports recommendations that delivery of an SSRI-exposed infant should take place in a facility equipped for neonatal resuscitation. Importantly, the target trial emulation used here is generalizable beyond SSRIs and offers a blueprint for evaluating the safety of other medications during pregnancy. As more longitudinal EHR data become available, this approach can advance drug safety research in this uniquely vulnerable population.

## Notes

Supplement 1. eTable 1. Definition of Variables Used in this Study (Data Dictionary) eTable 2. List of ICD-9 and ICD-10 Codes Used to Extract Suspected Cases of Congenital Anomalies for Further Validation Through Manual Chart Review eTable 3. Prevalence of Congenital Anomalies in the Periconception Cohort (total = 807 patients) eTable 4. Unadjusted and Inverse Probability Weighted Mean Difference and Odds Ratio of Adverse Birth Outcomes in the Early-Pregnancy Trial Among Infants of Women Who Continued SSRIs Compared With Women Who Discontinued SSRIs During Pregnancy eTable 5. Unadjusted and Inverse Probability Weighted Odds Ratio of Adverse Birth Outcomes in the Periconception Trial Among Infants of Women Who Continued SSRIs Compared With Women Who Discontinued SSRIs During Pregnancy eTable 6. Unadjusted and Inverse Probability Weighted Mean Difference and Odds Ratio of Adverse Birth Outcomes Among Infants of Women Who Continued SSRIs During Pregnancy (From the Early-Pregnancy Trial) Compared With Women Who Were Never Exposed to SSRIs During or Before Pregnancy eTable 7. Unadjusted and Inverse Probability Weighted Mean Difference and Odds Ratio of Adverse Birth Outcomes in the Early-Pregnancy Trial Sensitivity Analysis Using a 1-Year Prepregnancy Exposure Window, Comparing Continuation vs Discontinuation eTable 8. Unadjusted and Inverse Probability Weighted Odds Ratio of Adverse Birth Outcomes in the Periconception Trial Sensitivity Analysis Using a 1-Year Prepregnancy Exposure Window, Comparing Continuation vs Discontinuation eTable 9. Unadjusted and Inverse Probability Weighted Mean Difference and Odds Ratio of Adverse Birth Outcomes in the Early-Pregnancy Trial Sensitivity Analysis Using Inverse Probability of Censoring Weights to Account for Treatment Switching, Comparing Continuation vs Discontinuation eTable 10. Unadjusted and Inverse Probability Weighted Odds Ratio of Adverse Birth Outcomes in the Periconception Trial Sensitivity Analysis Using Inverse Probability of Censoring Weights to Account for Treatment Switching, Comparing Continuation vs discontinuation

Supplement 2. eAppendix 1. Eligibility Criteria and Exposure Ascertainment eAppendix 2. List of Non-SSRI Antidepressants and Anxiolytics for Exclusion Appendix 3. Method for Establishing and Validating Congenital Anomalies eFigure 1. Distribution of 1-Minute Apgar Scores Across SSRI Exposure Groups in the Early-Pregnancy Cohort eFigure 2. Distribution of 5-Minute Apgar Scores Across SSRI Exposure Groups in the Early-Pregnancy Cohort eFigure 3. Comparison of Primary and 1-Year Exposure Window Sensitivity Analyses for Neonatal Outcomes in the Early-Pregnancy Trial eFigure 4. Comparison of Primary and 1-Year Exposure Window Sensitivity Analyses for Neonatal Outcomes in the Periconception Trial eFigure 5. Comparison of Primary and Treatment-Switching Sensitivity Analyses for Neonatal Outcomes in the Early-Pregnancy Trial eFigure 6. Comparison of Primary and Treatment-Switching Sensitivity Analyses for Neonatal Outcomes in the Periconception Trial eReferences.

Supplement 3. Data Sharing Statement
