# Supplementary material for: Neonatal Outcomes Following Selective Serotonin Reuptake Inhibitor Use During Pregnancy
Source: JAMA Netw Open. 2026 Jul 13;9(7):e2622790. doi: 10.1001/jamanetworkopen.2026.22790 (PMC13366201; doi:10.1001/jamanetworkopen.2026.22790)
Supplement: Supplement 2. — eAppendix 1. Eligibility Criteria and Exposure Ascertainment eAppendix 2. List of Non-SSRI Antidepressants and Anxiolytics for Exclusion Appendix 3. Method for Establishing and Validating Congenital Anomalies eFigure 1. Distribution of 1-Minute Apgar Scores Across SSRI Exposure Groups in the Early-Pregnancy Cohort eFigure 2. Distribution of 5-Minute Apgar Scores Across SSRI Exposure Groups in the Early-Pregnancy Cohort eFigure 3. Comparison of Primary and 1-Year Exposure Window Sensitivity Analyses for Neonatal Outcomes in the Early-Pregnancy Trial eFigure 4. Comparison of Primary and 1-Year Exposure Window Sensitivity Analyses for Neonatal Outcomes in the Periconception Trial eFigure 5. Comparison of Primary and Treatment-Switching Sensitivity Analyses for Neonatal Outcomes in the Early-Pregnancy Trial eFigure 6. Comparison of Primary and Treatment-Switching Sensitivity Analyses for Neonatal Outcomes in the Periconception Trial eReferences. [file jamanetwopen-e2622790-s002.pdf]

# Supplemental Online Content

Aref L, Hughey JJ, Shirazi S, Sucre JMS, Bastarche L. Neonatal outcomes following selective serotonin reuptake inhibitor use during pregnancy. *JAMA Netw Open*. 2026;9(7):e2622790. doi:10.1001/jamanetworkopen.2026.22790

eAppendix 1. Eligibility Criteria and Exposure Ascertainment
eAppendix 2. List of Non-SSRI Antidepressants and Anxiolytics for Exclusion
Appendix 3. Method for Establishing and Validating Congenital Anomalies
eFigure 1. Distribution of 1-Minute Apgar Scores Across SSRI Exposure Groups in the Early-Pregnancy Cohort
eFigure 2. Distribution of 5-Minute Apgar Scores Across SSRI Exposure Groups in the Early-Pregnancy Cohort
eFigure 3. Comparison of Primary and 1-Year Exposure Window Sensitivity Analyses for Neonatal Outcomes in the Early-Pregnancy Trial
eFigure 4. Comparison of Primary and 1-Year Exposure Window Sensitivity Analyses for Neonatal Outcomes in the Periconception Trial
eFigure 5. Comparison of Primary and Treatment-Switching Sensitivity Analyses for Neonatal Outcomes in the Early-Pregnancy Trial
eFigure 6. Comparison of Primary and Treatment-Switching Sensitivity Analyses for Neonatal Outcomes in the Periconception Trial
eReferences.

This supplemental material has been provided by the authors to give readers additional information about their work.

© 2026 Aref L et al. *JAMA Network Open*.

## eAppendix 1. Eligibility Criteria and Exposure Ascertainment

To emulate the target trial's eligibility criteria, we used structured medication data from the OMOP drug exposure table. Each medication entry was linked to an exposure date, which we translated into timing relative to the estimated date of conception (EDC).

The OMOP drug exposure table includes three types of medication records:

1. **Inpatient medication orders**

2. **Outpatient prescriptions written by a provider**

3. **Medication list entries**, typically based on patient self-report at a clinical visit

Only inpatient orders and outpatient prescriptions provide timestamps that reliably reflect when a medication was initiated. In contrast, medication list entries are dated by when they were entered into the record, which may not correspond to the actual start date of use. For this reason, we restricted inclusion to individuals with evidence of SSRI exposure through either an inpatient order or an outpatient prescription during the two years before EDC.

## eAppendix 2. List of Non-SSRI Antidepressants and Anxiolytics for Exclusion

Pregnancies with any record of exposure to the following medications that treat anxiety or depression were excluded:

Bupropion, Trazodone, Duloxetine, Mirtazapine, Venlafaxine, Amitriptyline, Nortriptyline, Desvenlafaxine, Doxepin, Imipramine, Clomipramine, Vortioxetine, Tranylcypromine, Desipramine, Vilazodone, Nefazodone, Maprotiline, Alprazolam, Lorazepam  Buspirone, Labetalol, Diazepam, Clobazam, Oxazepam, Clorazepate, Chlordiazepoxide.

## eAppendix 3. Method for Establishing and Validating Congenital Anomalies

To establish and validate congenital anomaly outcomes, we identified infants with at least one relevant ICD-9 or ICD-10 code recorded within the first 30 days of life, followed by manual chart review to confirm the presence of anomalies. ICD codes were selected using congenital anomaly groupings from PhecodeX[1], a standardized system for aggregating ICD codes for research (see Appendix Table 2 for code groupings). To improve specificity, we excluded commonly encountered minor anomalies, including parasacral dimple, congenital non-neoplastic nevus, skin tag, accessory nipple, lip/tongue tie, undescended testis, congenital hydrocele, patent foramen ovale (PFO), and patent ductus arteriosus (PDA). Infants without a qualifying ICD code in the first 30 days were presumed not to have congenital anomalies and were not reviewed. Multiple congenital anomalies were defined as having anomalies in two or more organ systems.

© 2026 Aref L et al. *JAMA Network Open*.

## eFigure 1. Distribution of 1-Minute Apgar Scores Across SSRI Exposure Groups in the Early-Pregnancy Cohort

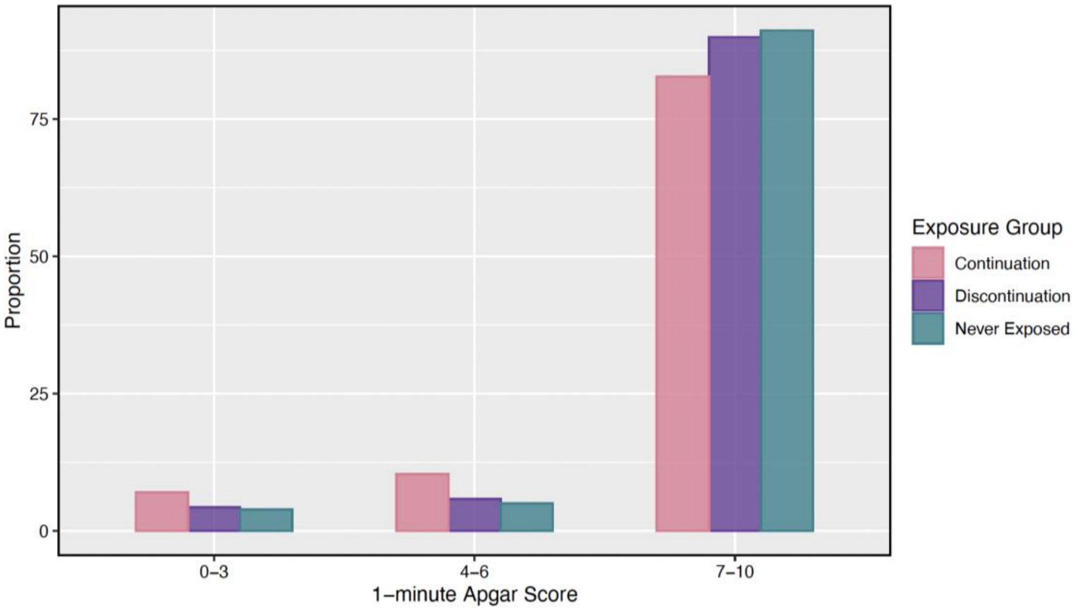


Scores are grouped into clinical categories: 0–3 (critically low), 4–6 (moderately low), and 7–10 (normal).

## eFigure 2. Distribution of 5-Minute Apgar Scores Across SSRI Exposure Groups in the Early-Pregnancy Cohort

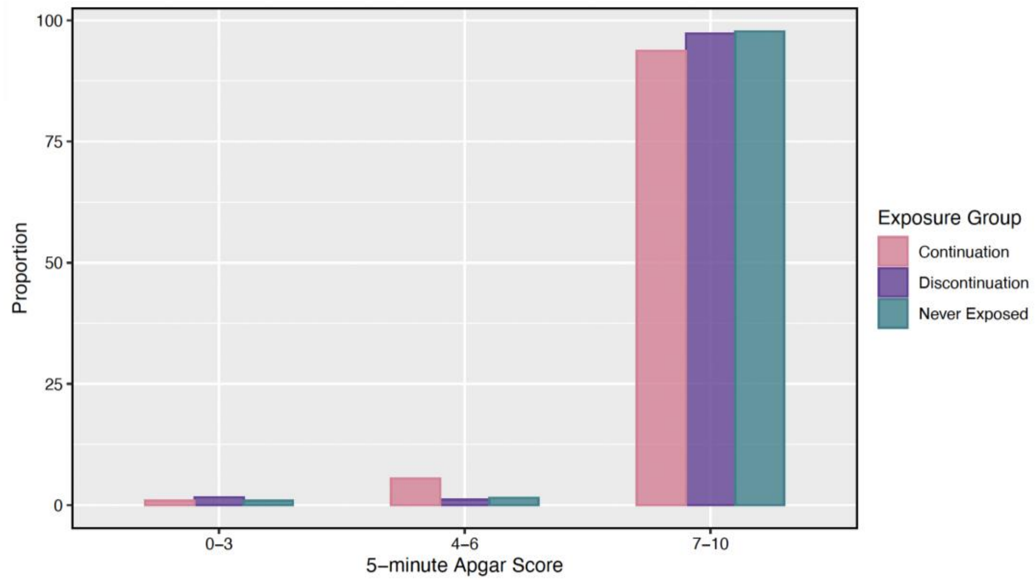


Scores are grouped into clinical categories: 0–3 (critically low), 4–6 (moderately low), and 7–10 (normal).

© 2026 Aref L et al. *JAMA Network Open*.

## eFigure 3. Comparison of Primary and 1-Year Exposure Window Sensitivity Analyses for Neonatal Outcomes in the Early-Pregnancy Trial

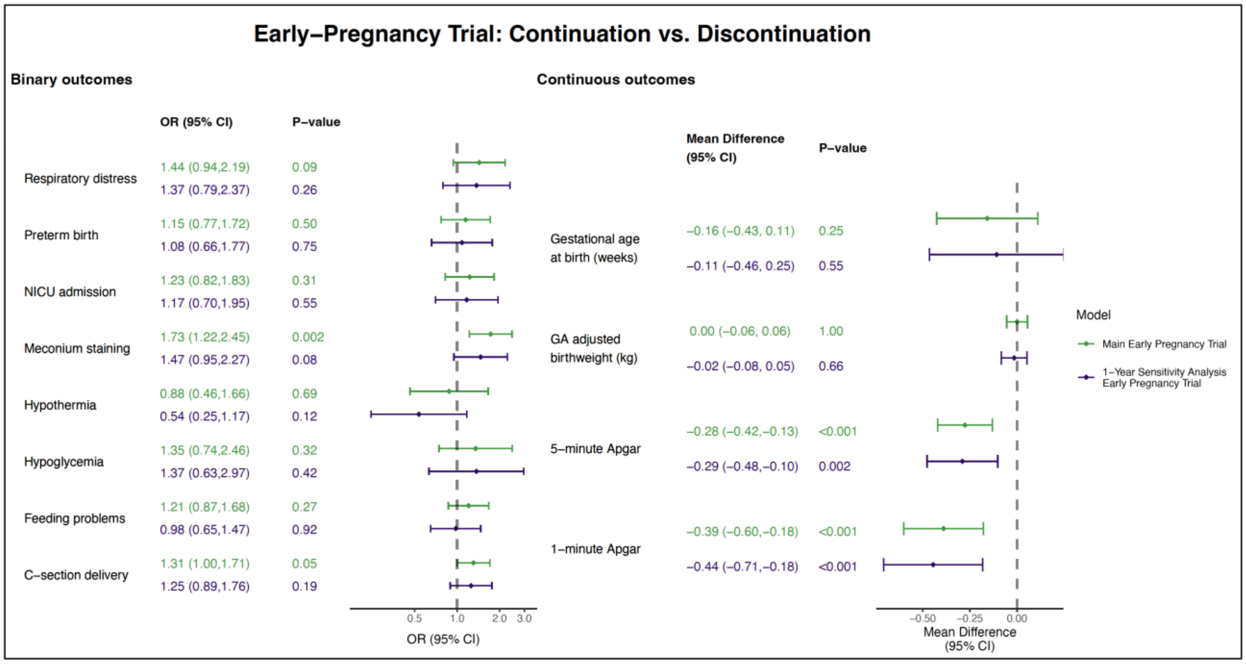


Odds ratios and mean differences for neonatal outcomes in the early-pregnancy trial, comparing continuation vs discontinuation (reference). Results from the primary analysis and the sensitivity analysis using a 1-year pre-pregnancy exposure window are shown, with cohorts distinguished by color. Note: PPHN was excluded due to wide confidence intervals, which affected interpretability of the plot.

## eFigure 4. Comparison of Primary and 1-Year Exposure Window Sensitivity Analyses for Neonatal Outcomes in the Periconception Trial

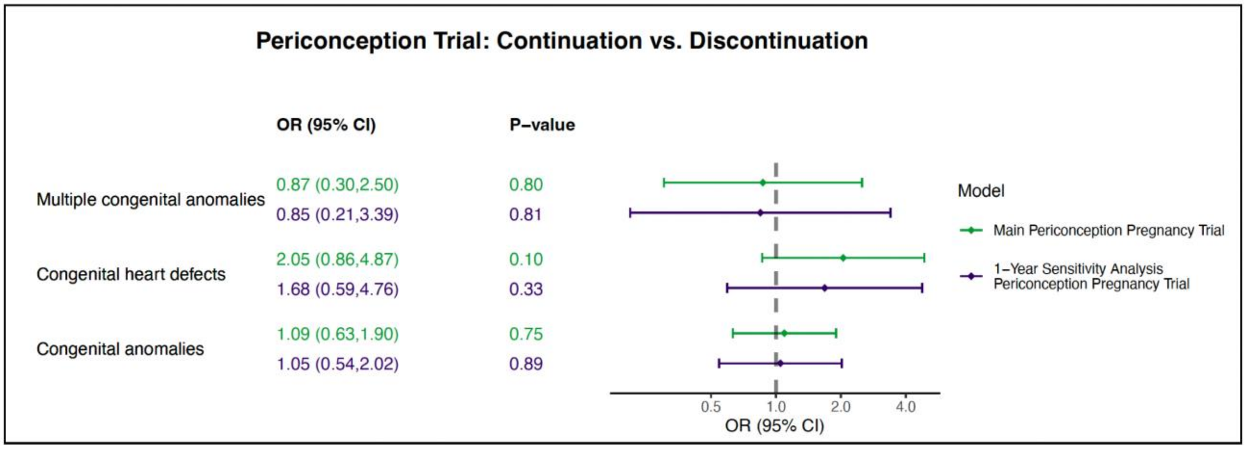


Odds ratios for neonatal outcomes in the periconception trial, comparing continuation vs discontinuation (reference). Results from the primary analysis and the sensitivity analysis using a 1-year pre-pregnancy exposure window are shown, with cohorts distinguished by color.

© 2026 Aref L et al. *JAMA Network Open*.

## eFigure 5. Comparison of Primary and Treatment-Switching Sensitivity Analyses for Neonatal Outcomes in the Early-Pregnancy Trial

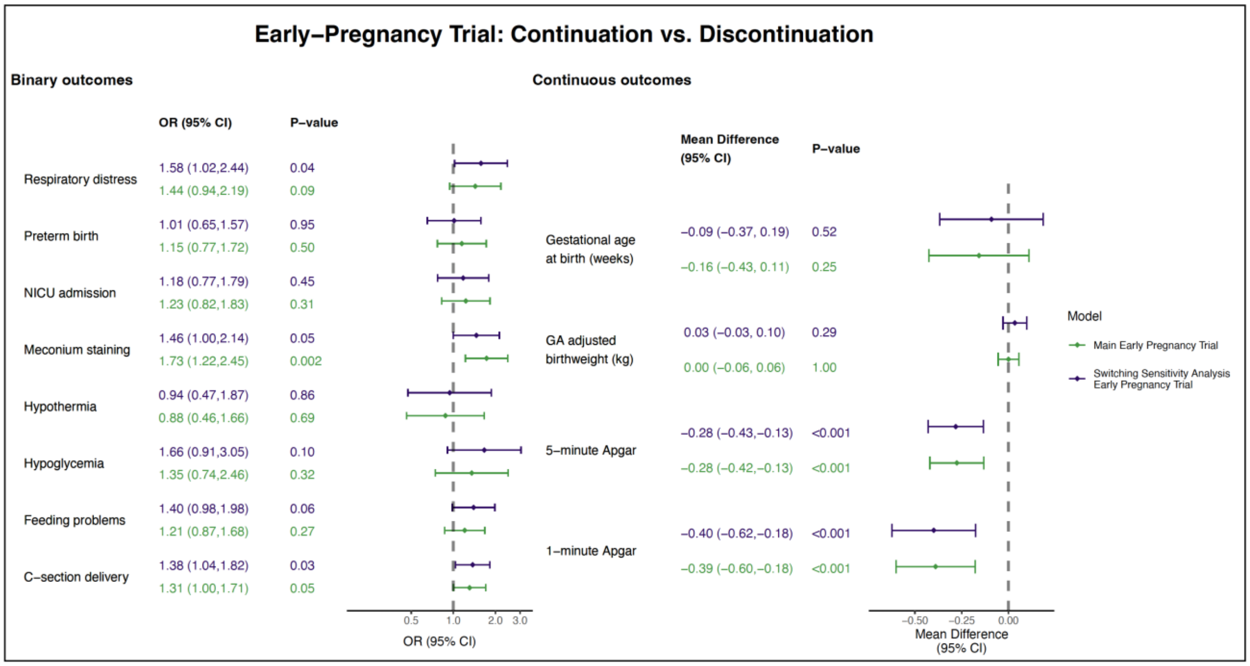


Odds ratios and mean differences for neonatal outcomes in the early-pregnancy trial, comparing continuation vs discontinuation (reference). Results from the primary analysis and the sensitivity analysis accounting for treatment switching using inverse probability of censoring weights are shown, with cohorts distinguished by color. Note: PPHN was excluded due to wide confidence intervals, which affected interpretability of the plot.

## eFigure 6. Comparison of Primary and Treatment-Switching Sensitivity Analyses for Neonatal Outcomes in the Periconception Trial

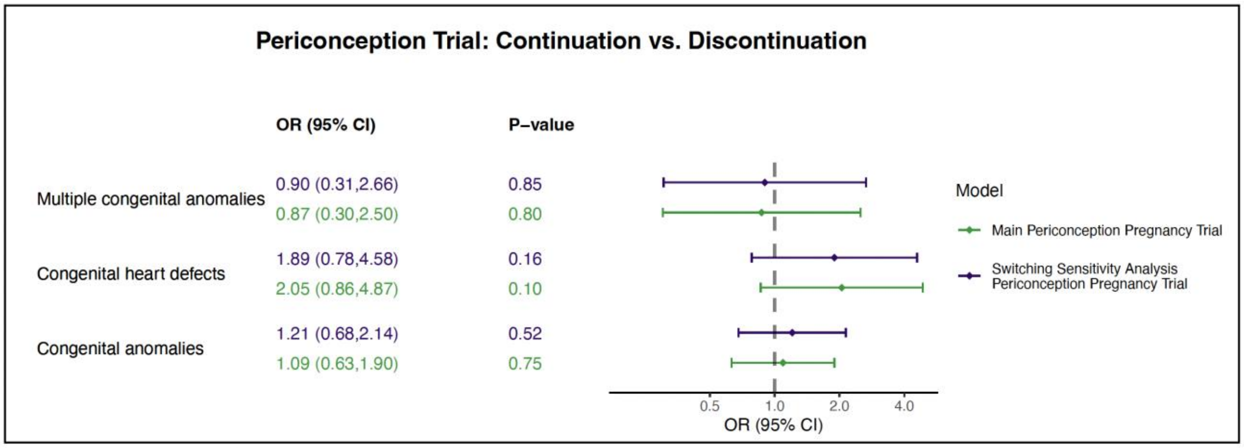


Odds ratios for neonatal outcomes in the periconception trial, comparing continuation vs discontinuation (reference). Results from the primary analysis and the sensitivity analysis accounting for treatment switching using inverse probability of censoring weights are shown, with cohorts distinguished by color.

© 2026 Aref L et al. *JAMA Network Open*.

## eReferences.

1. Shuey MM, Stead WW, Aka I, et al. Next-generation phenotyping: introducing phecodeX for enhanced discovery research in medical phenomics. *Bioinformatics*. 2023;39(11):btad655. doi:10.1093/bioinformatics/btad655

© 2026 Aref L et al. *JAMA Network Open*.
