# Supplementary material for: Neonatal Outcomes Following Selective Serotonin Reuptake Inhibitor Use During Pregnancy
Source: JAMA Netw Open. 2026 Jul 13;9(7):e2622790. doi: 10.1001/jamanetworkopen.2026.22790 (PMC13366201; doi:10.1001/jamanetworkopen.2026.22790)
Supplement: Supplement 3. — Data Sharing Statement [file jamanetwopen-e2622790-s003.pdf]

Data Sharing Statement

Aref. Neonatal Outcomes Following Selective Serotonin Reuptake Inhibitor Use During Pregnancy. *JAMA Netw Open*. Published July 13, 2026.
doi:10.1001/jamanetworkopen.2026.22790

Data
Data available: No

Additional Information
Explanation for why data not available: Due to the sensitive nature of the dataset, we cannot share row level data for this study.
